# The Impact of Plasmodium Berghei Exposure In-utero on Neurobehavioral Profile in Mice

**DOI:** 10.32598/bcn.9.10.95

**Published:** 2019-03-01

**Authors:** Akhabue Keneth Okojie, Khalid Rauf, Eghosa Iyare

**Affiliations:** 1. Reproductive and Developmental Programming Research Group, Department of Physiology, Enugu Campus, University of Nigeria, Enugu, Nigeria.; 2. Department of Pharmacy, COMSATS Institute of Information Technology, Abbottabad, Pakistan.; 3. Department of Physiology, Edo University Iyamho, Uzairue, Edo State, Nigeria.

**Keywords:** Plasmodium berghei, Malaria, In-Utero, Anxiety, Obsessive-Compulsive Disorder

## Abstract

**Introduction:**

The World Health Organization estimates that about 25 million pregnant mothers are currently at risk for malaria, and that malaria accounts for over 10,000 maternal and 200,000 neonatal deaths per year. The current hypothesis of early life programming supports the premise that many developmental delay and disorders may have their origin In-utero. Therefore, the current study aimed at evaluating the possible impact of experimental malaria exposure In-utero on neurobehavioral profile in mice offspring.

**Methods:**

Pregnant mice were intraperitoneally infected on gestational day 13 with 1.02×10^5^ infected red blood cells. Pregnant mice (both infected and uninfected) were allowed to deliver and the offspring were monitored up to postnatal day 42 when anxiety-like, Obsessive-Compulsive Disorder (OCD), and locomotor activity were evaluated using elevated plus maze, marble burying, and Open Field Test, respectively.

**Results:**

The current study showed that maternal infection with Plasmodium berghei resulted in an interesting behavior in offspring characterized by increased anxiety-like and OCD behaviors. Locomotor activity was however not affected.

**Conclusion:**

It may be concluded that In-utero exposure to experimental malaria in mice causes behavioral changes.

## Highlights

- In-Utero plasmodium berghei infection induces anxiety-like behavior in the offspring.
- In-Utero plasmodium berghei infection induces obsessive-compulsive disorder in the offspring.
- In-Utero plasmodium berghei infection does not have any effect on locomotor activity in the offspring.

## Plain Language Summary

The World Health Organization estimates that about 25 million pregnant mothers are currently at risk of inflicting malaria. Also, malaria accounts for over 10000 maternal and 200000 neonatal deaths per year mainly in regions where malaria infection is very high. First-time pregnant women could develop the clinical signs of malaria whereas females who had given birth before with greater immunity may be asymptomatic and therefore not seek treatment, leading to a greater placental parasite load. It could result in significant problems for the developing fetus, especially the brain of the fetus. Therefore, we used experimental malaria in pregnant animals to see how malaria will affect the behavior of offspring. We found out that baby of pregnant animals that had malaria exposure in the uterus, presented with different behavioral problems like anxiety, obsessive-compulsive disorder when the babies were 42 days old. These behavioral changes probably implicate that asymptomatic pregnant mother may potentially expose her fetus to the chronic disease.

## 1. Introduction

The World Health Organization reported that about roughly 25 million pregnant mothers are at risk for malaria, which accounts for more than 10,000 maternal and 200,000 neonatal deaths each year (Hartman, Rogerson, & Fischer, 2010). These figures could undervalue the role of malaria on maternal morbidity and mortality. A research in Mozambique on the causes of maternal death through autopsy evaluation found that about 10% of maternal deaths were directly attributed to malaria (Menendez et al., 2008). This implies that in regions in which malaria is endemic, it might directly lead to nearly 25% of maternal deaths. Malaria in pregnancy significantly contributes to perinatal morbidity and mortality. Infection is known to induce high levels of miscarriage, intrauterine passing, premature birth, low-birth-weight neonates, and neonatal death (Ofori et al., 2009).

Expecting mothers are three times more at risk for acute disease due to malaria infection in comparison with their non-expecting counterparts, and also have a mortality rate of nearly 50% due to acute disease (Monif & Baker, 2004). It is hypothesized that a vast majority of consequences in pregnancy outcomes results from two fundamental determinants: the immune compromised condition of pregnancy and placental sequestration of the infected erythrocytes. During pregnancy, sequestration of malaria infected Red Blood Cells (iRBC) occurs through the placenta and causes acute anemia as a consequence of the disease in the expecting mothers.

Interestingly, extreme level of placental infection is observed in gravid females with the maximum degree of immunity resulting in moderate maternal manifestation as well as an inordinate growth in fetal complications (Monif & Baker, 2004). Therefore, it might be hypothesized that though primigravidas could develop the clinical signs of malaria, females with greater immunity may be asymptomatic and therefore not seek treatment, leading to a greater placental parasite load. Fetal complications include the placental inflammation, maternal anemia, stillbirth, intrauterine growth restriction, and Low Birth Weight (LBW) neonates.

LBW, as a consequence of preterm delivery or fetal growth restriction, is associated with high risk of developmental setback in children (Allen, 2008; Procianoy, Koch, & Silveira, 2009). In addition, malnutrition and poverty are also considered as risk factors for developmental delay. However, many studies demonstrate that early exposure to diseases such as malaria might have significant deleterious effects on nervous system development. In-utero exposure to infections is associated with increased risk of neurodevelopmental disorders in offspring (O’Callaghan et al., 1994); Nevertheless, there is lack of information regarding the impact of malaria infection during pregnancy on neurobehavioral and cognitive functions of the babies exposed prenatally.

Recent studies thus suggest variables that interfere with the closely controlled In-utero environment may alter the normal nervous system developmental processes (Bilbo & Schwarz, 2009; Bale et al., 2010). Current hypothesis of early life programming buttresses the assumption that lots of developmental delay and ailments might have their source In-utero (Okojie et al., 2017; Iyare & Iyare 2006; Iyare & Adegoke 2008; Iyare & Iyare 2007; Bale et al., 2010).

Epidemiological studies also associate maternal infections with greater threat of developmental setback, schizophrenia, autism, and periventricular leukomalacia; a major cause of cognitive impairment in LBW babies (Ellman & Susser 2009). Murine models also show that offspring exposed to maternal infections In-utero experience long-term behavioral deficits including modified gene expression in the brain, altered neurotransmitter physiology, and regional cerebral atrophy (Shi, Fatemi, Sidwell, & Patterson, 2003; Nyffeler, Meyer, Yee, Feldon, & Knuesel, et al., 2006; Ozawa et al., 2006; Gilmore, Jarskog, Vadlamudi, & Lauder, 2004; Golan, Lev, Hallak, Sorokin, & Huleihel, 2005; Meyer et al., 2006), which were however not associated with congenital infection of the fetus. According to increased funding to fight both malaria and maternal mortality, the knowledge about In-utero malaria infection precisely alters offspring development and is vital in the efforts to improve maternal and perinatal health and suppress the spread of the preventable infectious diseases. Hence, the current study aimed at assessing the potential effect of experimental malaria exposure In-utero on neurobehavioral profile in mice offspring.

## 2. Methods

### 2.1. Animals and parasites

The BALB/c mice were obtained from National Institute of Health, Pakistan, and maintained in conventional housing at COMSATS Institute of Information Technology. Infection experiments were conducted in adult females with 10–12 weeks of age. Plasmodium berghei was a gift from Dr. Aina (Nigeria Institute for Medical Research, Lagos, Nigeria). The iRBC used in the experimental infections were obtained from in vivo passage in BALB/c mice, when the percentage of iRBC was roughly 10%. Thin blood smears were collected daily and stained with Giemsa to monitor parasitemia. All animals were fed with normal rat chow and all procedures conformed to the national guidelines on animal experimentation and welfare and were approved by Comsats Institute of Information Technology.

### 2.2. Gestation timing and pregnancy monitoring

Presence of vaginal plug and increase of body weight were collectively utilized to determine the time of pregnancy, according to Freyre et al. (2006) two female mice were placed together with a male mouse for two days, and the presence of vaginal plug was controlled each morning. The day of locating the vaginal plug was regarded as Gestation Day one (GD1) and pregnancy development was monitored every day by weighing the females. Successful fertilization was proven from G10 to G13 once the female mouse had a mean weight gain of 3–4 g. Therefore, weight increase was taken as the indication of pregnancy and sudden weight loss as the index of pregnancy loss or interference.

### 2.3. Pregnancy experimental infection

Pregnant mice were Intraperitoneally (IP) inoculated on GD13 with 1.02×10^5^ iRBC, and parasitemia was recorded every day. The model employed in the current study was predicated on a formerly supported murine model of Malaria in Pregnancy (MIP), which reproduced key pathogenic factors of MIP (Neres, Marinho, Gonçalves, Catarino, & Penha-Gonçalves, 2008). A lower dose of inoculum 10^5^ P. berghei infected erythrocytes, compared with a 10^6^ dose utilized in the current study to get rid of a very low birth weight phenotype and raise the amount of live births. Non-infected pregnant mice were used as controls. Pregnant mice (both control and experimental) were permitted to deliver and their offspring were monitored up to the Postnatal Day (PND) 42. Since P. berghei is deadly in BALB/c mice, foster mothers were utilized in the newborn post-natal follow-up studies. Consequently, progenies both from the infected mothers and control ones were additionally transferred to the foster mothers to prevent weight bias due to differential maternal nourishment. The progenies were weighed each day.

### 2.4. Neurobehavioural assay

#### 2.4.1. Open Field Test (OFT)

Each mouse was separately positioned in the middle of a white acrylic cage (30×30×15 cm) and was allowed to explore it for 30 minutes. In this time period, total distance travelled, number of rearing (standing on hind legs with paws pushed against the walls of the stadium), and episodes of grooming were recorded. At the conclusion of testing, the amount of fecal boli was determined and the cage was cleaned using a 10% ethanol solution. In this protocol, the locomotor activity was signified by total distance travelled in the cage, while the vertical display was assigned by the number of rearing. In regards to defecation in male mouse, under certain conditions, this parameter seemed to signify not an emotional reaction but a kind of scent marking (Archer, 1973; Walsh & Cummins 1976).

#### 2.4.2. Marble Burying Test (MBT)

Both in laboratory and natural environments, mice use accessible bedding material to cover obnoxious sources of distress existing in their home surroundings (Archer, Fredriksson, Lewander, & SÖDerberg, 1987). Within this protocol, 10 glasses of marble were equally dispersed in the plastic cage (35×50×35 cm) in the presence of a mouse. It was estimated that after 30 minutes, about two-thirds of the marbles were concealed.

#### 2.4.3. Elevated Plus Maze Test (EPMT)

Elevated plus maze reflects a conflict between the rodents' preference for protected areas and their innate motivation to explore novel environments (Pellow, Chopin, File, & Briley, 1985). An apparatus consisting of four arms (30×5 cm) was placed 50 cm above the ground. Two opposite arms were delimited by acrylic vertical walls; whereas the other two opposite arms had unprotected edges (open arms). Mice were placed in the center of maze and allowed to move freely for five minutes. During this period, the cumulative time and frequency of entries into open and closed arms were recorded. An arm entry was defined as the entry of the four paws into an arm. Then, the percentage of entries in open arms, percentage of the time spent in open arms, and the time spent in central platform were calculated. The anxiety-like behavior is linked to these last parameters.

Elevated plus maze reflects a battle between the rodents` desire for secure areas and their inborn motivation to search novel surroundings (Pellow et al., 1985). The apparatus consisting of four arms (30×5 cm) was placed 50 cm above the ground. The two opposite arms were delimited by acrylic vertical walls; whereas the other two arms had unprotected borders (open arms). Mice were placed in the middle of maze and permitted to move freely for five minutes. In this time, the cumulative frequency and time of entries into open and closed arms were recorded. An arm entrance was set as the entrance of four paws to an arm. Afterward, the proportion of admissions in open arms, portion of time spent in open arms and also the time spent in the central platform were computed. The anxiety-like behavior is connected to those last parameters.

### 2.5. Statistical analysis

All results are presented as a Means±Standard Error of Mean (SEM). Intergroup comparisons were evaluated with the Student unpaired-test. Differences were considered statistically significant when P<0.05. GraphPad Prism 5 (La Jolla, CA) was used for all statistical analyses.

## 3. Results

### 3.1. Blood parasiteamia

**Figure 1.**
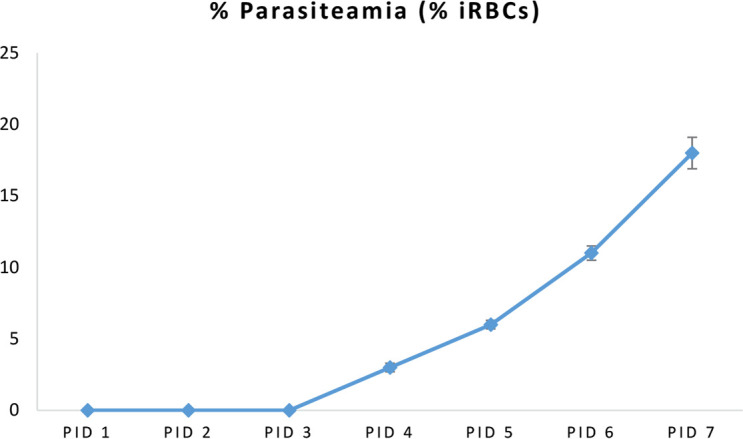
Maternal parasitaemia (GD14-20, n=7) shown as the percent of iRBCs

It was observed that exposure to P. beghei during pregnancy from GD13 led to gradual increase in the percentage of iRBCs as shown in Figure 1.

### 3.2. Open Field Test

**Figure 2.**
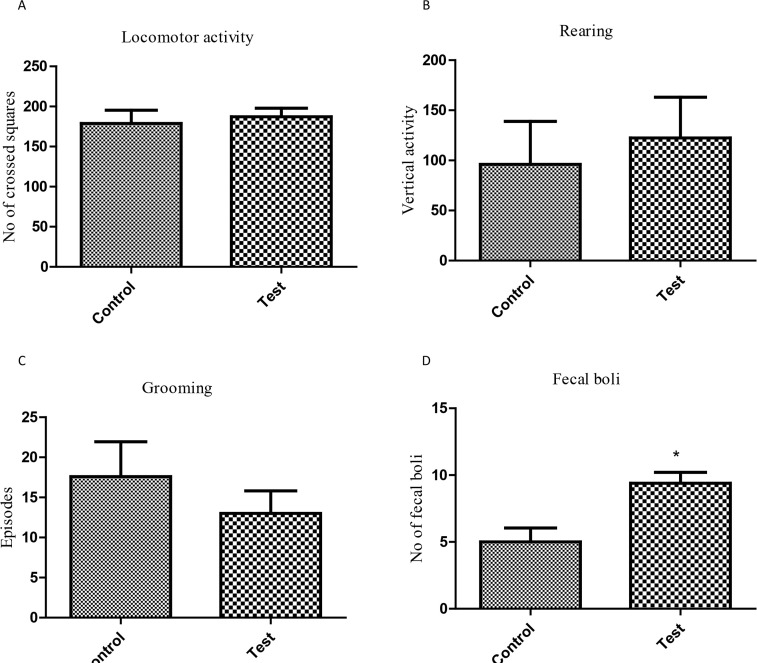
Evaluation of locomotor activity and anxiety-like behavior in open-field test in the offspring of mouse exposed to P. berghei In-utero a) Total distance covered; b) Rearing behavior; c) Grooming behavior; d) Number of fecal boli; Each bar represents Mean±SEM of the data (n=5); ^*^P<0.05 compared with the control.

In this test, no significant difference was observed in locomotor (P=0.6803) and vertical activities (P=0.6671) between the control and experimental groups. Likewise, there was also no difference in episodes of grooming (P=0.4007). Nevertheless, the mice of P. berghei-exposed group had higher number of fecal pellets (5.0±1.1 vs. 9.4±0.8, P=0.0106, Figure 2).

### 3.3. Marble Burying Test (MBT)

**Figure 3.**
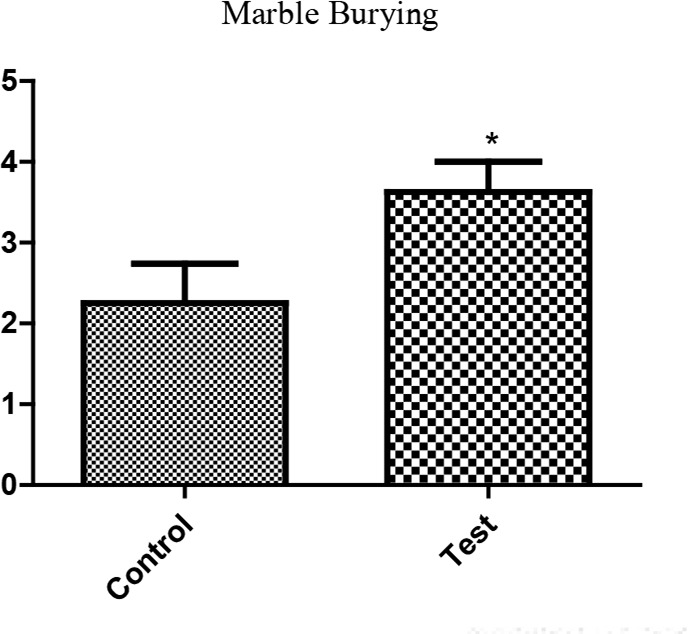
Assessment of anxiety-/OCD-like behavior in MBT in pups of mouse infected with P. berghei during pregnancy Each bar represents Mean±SEM of the data (n=8).

It was observed that exposure to P. berghei during pregnancy induced a significant increase in the amount of marbles buried in the experimental group, compared with that of the controls (2.3±0.5 vs. 3.6±0.4, P=0.043, Figure 3).

### 3.4. Elevated Plus Maze Test (EPMT)

**Figure 4.**
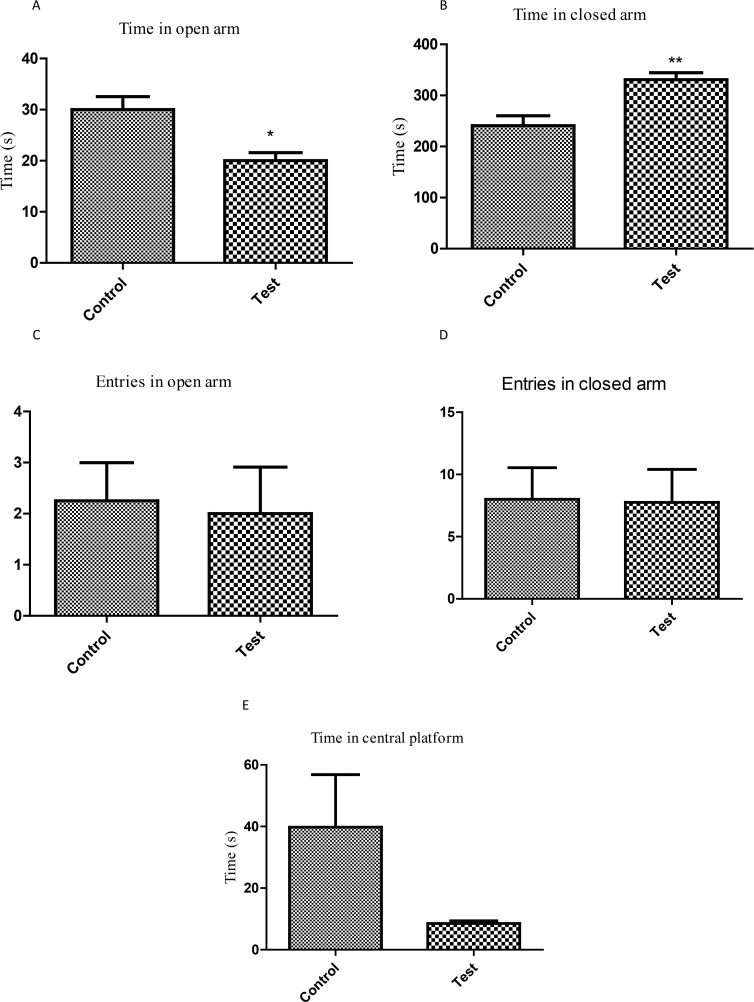
Evaluation of neurobehavioral profile in the offspring of mouse exposed to P. berghei infection In-utero using Elevated Plus Maze Test a) Time spent in open arms; b) Time spent in closed arms; c) Entries in open arms; d) Entries in closed arms; and e) Time spent in central platform; Each bar represents Mean±SEM (n=5); ^**^P<0.0095; ^*^P<0.0157 compared with controls.

With the Elevated Plus Maze Test, the group exposed to P. berghei spent different time in the closed arm (240.8±19.6 vs. 330.8±13.9 seconds, P=0.0095, Figure 4) and in the open arm (30.0±2.5 vs. 21.75±1.6 seconds, P=0.0157, Figure 4). There was no statistical difference in other anxiety-related parameters such as entries in closed arm (P=0.9481), entries in open arm (P=0.8394), or time in central platform (P=0.1169).

## 4. Discussion

Despite the fact that malaria infection during pregnancy is very common in sub-Sahara regions, there are very limited studies that demonstrate the effects of maternal infection with malaria in the offspring’s behaviors. McDonald et al. (2015) showed that In-utero exposure to maternal malaria infection could change the cognitive and neurological development of offspring. Results of the current study showed that maternal infection with P. berghei resulted in an interesting behavior of offspring characterized by increased anxiety-like and OCD behaviors.

The offspring exposed to P. berghei were neither congenitally infected nor presented with any phenotypic adverse outcomes associated with MIP. Malaria during pregnancy is marked with the aggregation of iRBCs and monocytes/macrophages in the intervillous space, leading to a specific local immune response in the placenta (Umbers, Aitken, & Rogerson, 2011). The current study results showed a persistent, but gradual increase in the Parasitized Erythrocytes (PEs). Nonetheless, the 10^5^ inoculum was correlated with reduced maternal peripheral parasiteamia compared with that of the Neres et al. (2008) using a dose of 10^6^ PEs. These results suggested that similar to humans, pregnant mice show increased susceptibility to malaria infection that may affect their progeny or compromise pregnancy, since the current study observed a persistent, but gradual increase in PEs.

The current study observed that P. berghei infection during pregnancy encouraged anxiogenic response in Open Field Test (increased defecation and rearing), and in Elevated Plus Maze Test (reduced percentage of time in open arms and increased time in closed arm). It is logical that the above observations could be a result of distortion of the neuromodulatory systems within the cerebral cortex known to influence behavior. An obvious anxiogenic effect of In-utero malaria infection is that it provides the opportunity to assume that it is associated with a rapid decrease in the amount of serotonin (5-HT) in different cerebral structures. It was previously observed that the case of the cerebral serotonergic system firmly corresponded with the level of stress in rodents and humans (Montegomery, 1991; Griebel, Moreau, Jenck, Misslin, & Martin, 1994).

Grooming activity non-significantly reduced in the experimental group compared with that of the control. Previous studies showed that selective agonists of dopamine D1 and D5 receptors evoked the escalation of grooming behavior, while D2 receptor agonists considerably curtailed this type of behavioral activity (Briley, Chopin, & Moret, 1990; Ferrari, Pelloni, & Giuliani, 1992). Thus, a selective action to some extent as a result of In-utero malaria infection on particular kinds of dopamine receptors should be presumed.

The current study results also showed that experimental malaria infection in mice offspring resulted in increased marble burying, which is a test to evaluate OCD in rodents. It might also reflect the elements of defensive behavior; rodents try to stop predators such as snakes from penetrating their burrows by digging soil and kicking it toward the intruder. Defensive burying is a renowned paradigm to measure anxiety (Pinel & Treit, 1978). The burrowing paradigm proved a sensitive assay to track the growth of prion disease in mice (Deacon, Raley, Perry, & Rawlins, 2001; Guenther, Deacon, Perry, & Rawlins, 2001).The neuronal circuitry of the specific behavior is not yet definitely understood. Thomas et al. (2009) claimed that marble burying reflects a persistent and perseverative behavior over novelty-induced anxiety. The hippocampus and septum are likely to be significant, since hippocampal cytotoxic lesions largely reduce burrowing (Deacon, Croucher, & Rawlins, 2002), and medial prefrontal cortex lesions cause a more compact reduction (Deacon, Penny, & Rawlins, 2003). Some studies also demonstrated that lots of agents including psychostimulants inhibit marble burying (Millan, Girardon, Mullot, Brocco, & Dekeyne, 2002; Wicke & Gross 2005).

Therefore, the current study results demonstrated that experimental In-utero malaria infection possessed a significant modulatory behavioral potential. Experimental In-utero malaria infection resulted in anxiogenic, escape, and OCD behaviors in mice offspring. These behavioral shifts probably show that the implications of asymptomatic pregnant mother may potentially expose her fetus to the chronic disease. It is therefore imperative that all pregnant mothers in malaria endemic regions be screened for malaria parasite and given safe anti-malaria drugs such as prophylaxis. At the same time, the probable mechanisms underlying how In-utero malaria infection affects the cerebral modulatory systems (this kind of action based on the available information, appears to be quite likely) may need specific thorough investigations.

## Ethical Considerations

### Compliance with ethical guidelines

The study protocol was approved by Ethics Committee of Department of Pharmacy, CIIT, Abbottabad Campus (Ethical code: PHM.ETH/FA.16-1116-CIIT-ATD).
